# Clinically Suspected Exercise-Induced Myocarditis in a Patient With Vascular Ehlers-Danlos Syndrome

**DOI:** 10.7759/cureus.29689

**Published:** 2022-09-28

**Authors:** Rishi Kalia, Ravi Kalia, Merly Cubelo, Joshua Musih, Jesal Popat

**Affiliations:** 1 Dr. Kiran C. Patel College of Osteopathic Medicine, Nova Southeastern University, Fort Lauderdale, USA; 2 Interventional Cardiology, Florida Medical Clinic, Tampa, USA

**Keywords:** vascular ehlers danlos syndrome, myocarditis, exercised induced myocarditis, ehlers danlos syndrome type iv, ehlers danlos syndrome

## Abstract

Ehlers-Danlos syndrome (EDS) is an inherited condition that affects connective tissue structures throughout the body. EDS type IV is a specific subset of EDS that targets vascular structures. Patients with EDS type IV can have a wide range of vascular disorders such as myocarditis and are advised to refrain from participating in contact sports and high-intensity physical exercises to avoid life-threatening cardiovascular conditions such as arterial dissection or rupture. In this report, we present the case of an EDS type IV patient with myocarditis secondary to exercise. The patient received supportive treatment including beta-blockers, bed rest, and refraining from intense physical exertion.

## Introduction

Ehlers-Danlos syndrome (EDS) refers to a group of genetic disorders affecting connective tissue. Patients often present with characteristic features such as translucent, fragile skin, easy bruising, joint hypermobility, and other signs of connective tissue involvement. The syndrome has been classified into 10 heterogenous subtypes, and we focus on type IV in this report.

EDS type IV, also known as vascular Ehlers-Danlos syndrome (VEDS), leads to vascular complications commonly involving large and medium caliber arteries. VEDS accounts for approximately 5-10% of all cases of EDS and is a particularly dangerous subtype. It exhibits an autosomal-dominant pattern of inheritance and affects the COL3A1 gene coding for type III pro-collagen [1]. Patients with EDS type IV commonly present with vertebral and carotid artery dissection, organ rupture, and gastrointestinal perforations.

The dermatologic presentations of EDS type IV are variable, with acrogeria being present in most patients. These patients tend to have thin skin with a tight, parchment-like character and an apparent venous pattern. Bruising can also be present, and in some cases, it is particularly prominent, prompting a hematologic evaluation [1]. There is no consensus in the scientific literature on the exact nature of the vascular lesions in EDS type IV. However, most appear to be the result of congenitally fragile tissue, leading to false aneurysms and internal hemorrhage. The majority of the aneurysms that have been recorded are false aneurysms, with some patients presenting with real fusiform aneurysms [2].

Previous reviews have reported vascular rupture as the hallmark of VEDS and the main cause of lower survival rates in these patients. In one review of 220 patients, 103 died secondary to arterial rupture and half of the remaining deaths were secondary to organ rupture [3].

In some individuals, the clinical diagnosis of VEDS can be quite apparent, with the majority of patients diagnosed before 18 years of age. However, this is not the case for all patients, and a clinical and biochemical evaluation for EDS should be considered in young individuals with arterial or bowel rupture without an evident cause, especially in those with a family history of similar events.

## Case presentation

A 19-year-old female with a history of EDS type IV was admitted to the ED for chest pain, elevated troponin, and ventricular tachycardia (V-tach). The patient was an undergraduate student-athlete on the school rowing team. She had been working out after consuming an energy drink and rowing on the rowing machine at the school gym. The patient stated that she had been rowing harder than usual, and had begun to experience chest pain that radiated to her left side and left arm shortly afterward. An EKG was performed and the patient was noted to have T-wave inversion in inferolateral leads on EKG. High-sensitivity serum troponin I was elevated at 23 ng/ml. Subsequently, the patient was airlifted to a nearby ED for further evaluation.

On arrival at the next ED, the patient stated that she was feeling mild discomfort but still felt like herself. A pediatric transthoracic echocardiogram was ordered, detailing normal biventricular function and mild mitral valve regurgitation with an estimated ejection fraction of 65%. A CT angiogram (CTA) was subsequently ordered due to concerns for aortic dissection. The CTA demonstrated no dissection or coronary abnormalities. The decision was made to admit the patient to the pediatric ICU (PICU) for further management. In the PICU, the patient was placed on continuous cardiac monitoring and was noted to have a 10-beat run of V-tach. A consult with the cardiology team led to a recommendation of starting the patient on 25-mg metoprolol extended-release (ER) and a transfer to the pediatric cardiac ICU (PCICU). 

**Figure 1:**
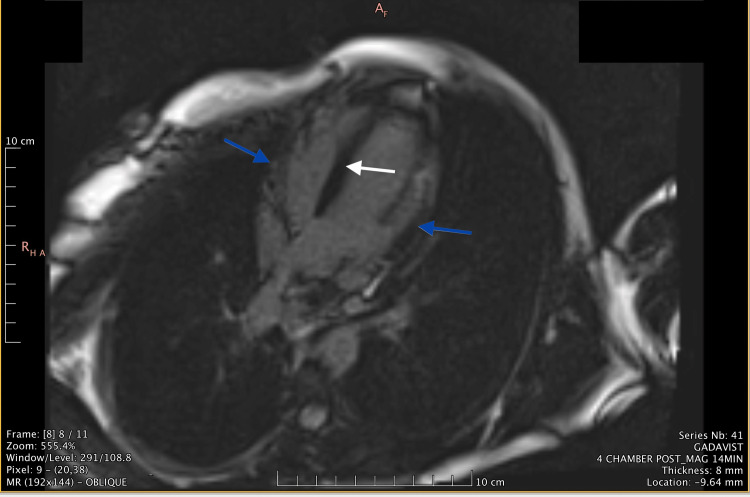
Oblique MRI image demonstrating late myocardial enhancement in a non-ischemic pattern within the septum and lateral wall characteristic of myonecrosis or scar Blue arrows indicate the areas of late myocardial enhancement in the lateral walls and white arrow indicates the areas of late myocardial enhancement in the septum MRI: magnetic resonance imaging

**Figure 2:**
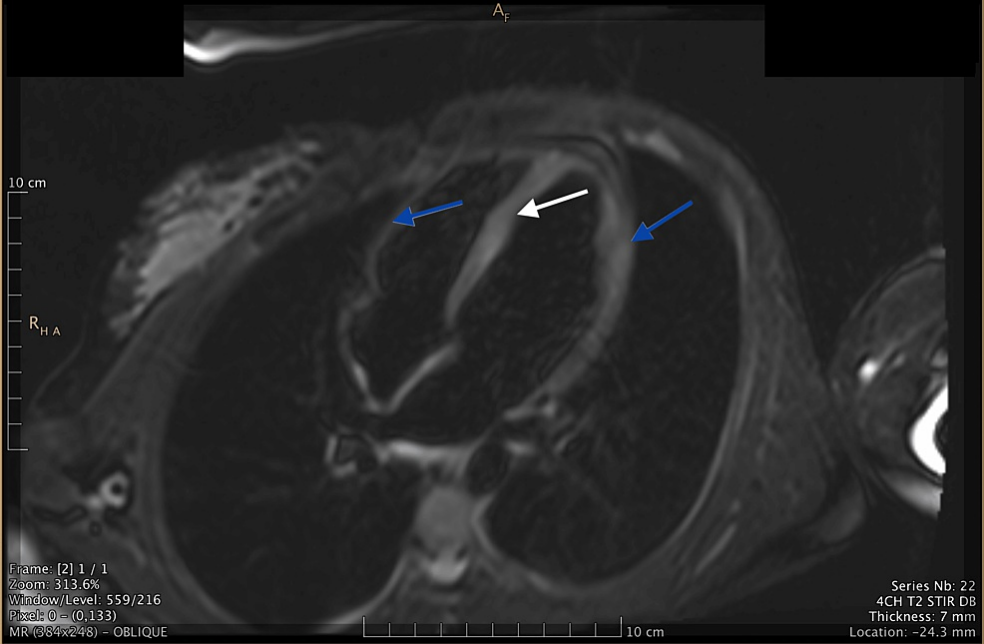
Oblique MRI image demonstrating T2 hyperintense myocardial enhancement of septal and lateral walls characteristic of myocardial edema Blue arrows indicate the areas of T2 hyperintense myocardial enhancement in the lateral walls and white arrow indicates the areas of T2 hyperintense myocardial enhancement in the septum MRI: magnetic resonance imaging

Upon arrival at the PCICU, the patient was continued on 25-mg metoprolol ER and monitored for recurrence of V-tach. No recurrence was noted. The patient’s troponin level peaked at 29.3 and was monitored daily until it was less than 2. A cardiac MRI was ordered, which showed abnormal delayed myocardial enhancement in a non-ischemic pattern within the septum and lateral wall, characteristic of myonecrosis or scarring (Figure 1). In addition, the cardiac MRI showed T2 hyperintense myocardial enhancement of the septal and lateral wall consistent with myocardial edema (Figure 2).

The patient was discharged after eight days with a plan to continue 25-mg metoprolol ER, PO, once daily, for 30 days. A follow-up appointment with pediatric cardiology and a nuclear stress test were scheduled in six weeks' time and the recommendation was made to adhere to a low level of physical activity for the next six weeks. The results of the nuclear stress test were unremarkable.

## Discussion

The patient in this case was diagnosed with an acute episode of clinically suspected exercise-induced myocarditis. The radiographic findings were consistent with two of the Lake Louise criteria for the assessment of myocarditis [3]. To establish a definitive diagnosis of myocarditis, an endomyocardial biopsy (EMB) would need to be completed including histologic (Dallas criteria) as well as immunohistochemical criteria according to the World Health Organization/International Society and Federation of Cardiology (WHO/ISFC) definition [4]. Having a pre-existing medical history of EDS type IV, this patient had a higher risk of developing cardiovascular abnormalities. The patient’s mother also had EDS type IV and passed away shortly after delivery due to aortic dissection. This was not the first occurrence of exercise-induced myocarditis in this patient. In 2020, physical overexertion had led to her experiencing crushing chest pain that radiated to the back. During the episode, the patient had also been admitted to the hospital with V-tach, elevated troponin levels, and evidence of myocarditis on cardiac MRI. Treatment at that time included six weeks of metoprolol 25-mg ER. The patient had recovered after a few months of precautionary measures including limiting physical activity.

During the latest occurrence in March of 2022, the patient presented in a similar fashion but was later advised to continue metoprolol 25-mg ER indefinitely as prophylaxis for the prevention of future events. The patient was advised to not engage in overexerting physical activities and eventually continue to complete all her activities of daily living.

The long-term prescription of beta-blockers in patients with EDS type IV as treatment has not been officially recommended, although there have been studies published regarding the efficacy of Celiprolol, a selective B1 receptor antagonist and B2 receptor partial agonist [2,5]. In terms of myocarditis, beta-blockers appear to play a cardioprotective role by lengthening diastole and are associated with a better clinical outcome [6,7]. Currently, there are no published cases describing EDS type IV-associated exercise-induced myocarditis in the medical literature, further reinforcing the importance of sharing this case with the medical community.

This case report will hopefully serve as a resource for medical professionals in the future when encountering similar patient presentations and are uncertain about the appropriate medical management for this novel diagnosis.

## Conclusions

EDS type IV is an inherited disease of connective tissue that specifically targets vascular structures. While its presenting features can vary, serious cardiac and vascular complications pose the greatest concern for clinicians. Our case report highlights a less commonly reported manifestation of VEDS, exercise-induced myocarditis.

The aim of our case report is to provide greater awareness about atypical presentations of VEDS. Although the causes of myocarditis may vary, VEDS should be considered in patients with a possible history of connective tissue disease. Further studies may help to elucidate the precise underlying mechanism of exercise-induced myocarditis in this patient population. Additionally, future reports may identify additional management strategies to better treat patients with VEDS-related myocarditis. The limitations of this case report include the sample size (only one individual), limited clinical data on long-term outcomes for the patient, and the lack of an established treatment protocol.
